# Repurposing AM404 for the treatment of oral infections by Porphyromonas gingivalis

**DOI:** 10.1002/cre2.65

**Published:** 2017-04-07

**Authors:** Evelien Gerits, Pieter Spincemaille, Kaat De Cremer, Katrijn De Brucker, Serge Beullens, Karin Thevissen, Bruno P.A. Cammue, Katleen Vandamme, Maarten Fauvart, Natalie Verstraeten, Jan Michiels

**Affiliations:** ^1^ Department of Microbial and Molecular Systems KU Leuven, Centre of Microbial and Plant Genetics Leuven Belgium; ^2^ Department of Laboratory Medicine University Hospitals Leuven Leuven Belgium; ^3^ Department of Plant Systems Biology VIB Ghent Belgium; ^4^ Department of Oral Health Sciences UZ Leuven, Restorative Dentistry‐KU Leuven, BIOMAT Leuven Belgium; ^5^ Department of Life Science Technologies imec, Smart Systems and Emerging Technologies Unit Belgium

**Keywords:** AM404, biofilms, N‐(4‐hydroxyphenyl)‐arachidonylamide, peri‐implantitis, periodontitis, Porphyromonas gingivalis

## Abstract

*Porphyromonas gingivalis* is a major pathogen involved in oral diseases such as periodontitis and peri‐implantitis. Management of these diseases typically includes mechanical debridement of the colonized surfaces followed by application of antiseptics or antibiotics. Disadvantages associated with the use of antiseptics and the growing worldwide problem of antibiotic resistance have necessitated the search for alternative agents. In this study, the antibacterial and antibiofilm properties of AM404, an active metabolite of paracetamol, were tested against *P*. *gingivalis* and other bacterial pathogens. The activity of AM404 was tested against 10 bacteria, including both oral and nonoral human pathogens. The minimal inhibitory concentration (MIC) of AM404 was determined by measuring optical density (OD) values. The minimum biofilm inhibitory concentration (MBIC) was detected by crystal violet staining. The activity of structural analogs of AM404 was tested by MIC determinations. The effect of AM404 on *P*. *gingivalis* biofilms formed on titanium disks as a model for dental implants was evaluated by colony forming unit counting. Potential cytotoxicity of AM404 towards HEK‐293 (human embryonic kidney cells), HepG2 (human hepatoma cells), IEC‐6 (rat intestinal cells), and Panc‐1 cells (pancreatic cancer cells) was assessed by 3‐(4,5‐dimethylthiazol‐2‐yl)‐2,5‐diphenyltetrazolium bromide assays. To get more insight in the mode of action of AM404, we used the fluorescent dyes N‐phenyl‐1‐napthylamine and SYTOX green to investigate outer and inner membrane damage of *P*. *gingivalis* induced by AM404, respectively. Of all tested pathogens, AM404 only inhibited growth and biofilm formation of *P*. *gingivalis*. Moreover, it showed potent activity against *P*. *gingivalis* biofilms formed on titanium surfaces. A structure–activity analysis demonstrated that the unsaturated carbon chain is essential for its antibacterial activity. Importantly, AM404 was not toxic towards the tested mammalian cells up to concentrations approaching 4× the MIC. Membrane damage assays using fluorescent probes N‐phenyl‐1‐napthylamine and SYTOX green revealed that membrane permeabilization presumably is the primary antibacterial mode of action of AM404. Collectively, our results suggest that AM404 has the potential to be used for the development of new drugs specifically targeting *P. gingivalis*‐related infections.

## 1 INTRODUCTION

*Porphyromonas gingivalis* is a Gram‐negative anaerobic bacterium identified as one of the major periodontal pathogens (How, Song, & Chan, 2016). It exerts its pathogenic effects via a subset of virulence factors including fimbriae and the production of cysteine proteinases, hemaglutinins, and lipopolysaccharides (Enersen, Nakano, & Amano, 2013). In addition, pathogenicity is enhanced by its ability to form biofilms on both biotic and abiotic surfaces, yielding protection against environmental stress factors such as antibiotics and host defense mechanisms (Costerton, Stewart, & Greenberg, 1999; De la Fuente‐Núñez, Reffuveille, Fernández, & Hancock, 2013).

*P. gingivalis* is recognized as a major pathogen involved in chronic inflammatory diseases such as periodontitis and peri‐implantitis, which result in the destruction of soft and hard tissues surrounding the teeth and dental implants, respectively (Ata‐Ali et al., 2011; Hajishengallis, Darveau, & Curtis, 2012; How et al., 2016; Mahato, Wu, & Wang, 2016). Recent studies have reported that the prevalence of periodontitis among U.S. adults is as high as 46% (Eke et al., 2015). In addition, the prevalence of peri‐implantitis is estimated to be about 20–56% for all implant patients, depending on the time frame under investigation (Mombelli, Müller, & Cionca, 2012; Zitzmann & Berglundh, 2008). These numbers are expected to increase substantially with the expected aging of the population.

Treatment of *P. gingivalis*‐mediated diseases usually includes surface debridement procedures and the administration of anti‐infective agents such as chlorhexidine or antibiotics (Herrera, Sanz, Jepsen, Needleman, & Roldán, 2002; Prakasam, Elavarasu, & Natarajan, 2012; Prathapachandran & Suresh, 2012; Quirynen, Teughels, & van Steenberghe, 2003; van Winkelhoff, 2012; Xajigeorgiou, Sakellari, Slini, Baka, & Konstantinidis, 2006). However, side effects are associated with the use of chlorhexidine, such as brown discoloration of the teeth, alteration in taste, supragingival calculus formation, and, more rarely, oral mucosal erosion and parotid swelling. Furthermore, this antiseptic is known to have a bitter taste (Eley, 1999; Kolahi & Soolari, 2006; Van Strydonck, Slot, Van der Velden, & Van der Weijden, 2012). In addition, some studies have reported a reduced efficacy of chlorhexidine in subgingival areas due to a low subgingival availability of the drug and the ability of *P. gingivalis* to release outer membrane vesicles that bind and inactivate chlorhexidine (Grenier, Bertrand, & Mayrand, 1995; Stabholz et al., 1993). Different classes of antibiotics have been used for the treatment of *P. gingivalis*‐related infections, including tetracyclines (tetracycline hydrochloride, minocycline, and doxycycline), macrolides (erythromycin), lincosamide (clindamycin), ß‐lactams (ampicillin and amoxicillin), and nitroimidazoles (metronidazole; Heitz‐mayfield & Mombelli, 2014; Kapoor, Malhotra, Grover, & Grover, 2012; van Winkelhoff, 2012). However, in recent years, the incidence of antibiotic‐resistant oral bacteria such as *P. gingivalis* has increased (Ardila, López, & Guzmán, 2010; Rams, Degener, & van Winkelhoff, 2014a; Rams, Degener, & van Winkelhoff, 2014b; Sweeney, Dave, Chambers, & Heritage, 2004), highlighting the need for new antibacterials targeting oral infections.

Recently, drug repurposing has emerged as a new strategy for the identification of new antibacterial agents (Rangel‐Vega, Bernstein, Mandujano‐Tinoco, Garcia‐Contreras, & Garcia‐Contreras, 2015). As the safety and pharmacokinetic profiles of market drugs are already known, production time and costs are much lower in comparison with de novo discovery of antibiotics (Ashburn & Thor, 2004; Chong & Sullivan, 2007; Rangel‐Vega et al., 2015). In an attempt to repurpose existing drugs as antibacterial agents, we recently screened the National Institutes of Health (NIH) clinical library against *P. gingivalis* (Gerits et al., 2017a,b). One of the hit compounds of the screening, AM404 (N‐(4‐hydroxyphenyl)‐arachidonylamide), an active metabolite of paracetamol, was further characterized in this study.

The first purpose of this study was to assess the antibacterial and antibiofilm activity of AM404 against *P. gingivalis* and other pathogens. In addition, cytotoxicity of AM404 was tested against different human cell lines. Finally, the antibacterial mode of action of AM404 was investigated.

## 2 MATERIAL AND METHODS

### 2.1 Bacterial strains and chemicals

*P. gingivalis* ATCC 33277, *Prevotella intermedia* ATCC 49046, *Fusobacterium nucleatum* ATCC 49256, and *Aggregatibacter actinomycetemcomitans* ATCC 43718 were routinely grown on agar (Blood agar base no. 2, Sigma‐Aldrich NV) supplemented with 5% horse blood (Horse Blood Defibrinated, Oxoid NV), hemin (5 μg/ml; Sigma) and menadione (1 μg/ml; Sigma) at 37 °C under anaerobic conditions (90% N_2_, 5% H_2_, and 5% CO_2_) using an Anoxomat AN2OP system (Mart Microbiology, Drachten, the Netherlands). *Streptococcus mutans* ATCC 25175, Staphylococcus aureus SH1000 (O'Neill, 2010), Staphylococcus epidermidis CH (Costerston, Marrie, & Cheng, 1985), Escherichia coli TG1 (Carter, Bedouelle, & Winter, 1985), *Pseudomonas aeruginosa* (Lee et al., 2006), and *Salmonella enterica* serovar Typhimurium SL1344 (Hoiseth & Stocker, 1981) were grown routinely on solid trypticase soy broth (TSB, Becton Dickinson Benelux) containing 1.5% agar at 37 °C. AM404, arvanil, 4′‐hydroxystearanilde, and R(+)‐methanandamide were purchased from Sigma‐Aldrich and stock solutions of 20 mM were prepared in dimethyl sulfoxide (DMSO).

### 2.2 Antibacterial assays

The minimal inhibitory concentration (MIC) of AM404 and its analogs were evaluated in TSB as described before (Liebens et al., 2014). To determine the minimal bactericidal concentration (MBC), 10 μl aliquots were taken from the wells of the MIC assay that did not show bacterial growth and were plated onto blood agar plates. After incubation of the plates, the MBC was determined as the lowest concentration of AM404 for which no colony‐forming units (CFUs) were observed.

### 2.3 Antibiofilm assays

The ability of AM404 to inhibit biofilm formation of *P. gingivalis* was determined using the Calgary Biofilm Device (Nunc‐Immuno TSP, VWR International) as described previously, with some modifications (Janssens et al., 2008). A bacterial suspension was prepared by diluting an overnight culture of *P. gingivalis* 1/10 in TSB. Twofold serial dilutions of AM404 in bacterial suspension (150 μl) were prepared in the wells of 96‐well microtiter plates. Thereafter, the peg lid of the Calgary Biofilm Device was placed on top of the microtiter plate and the biofilms were allowed to grow on the pegs for 72 hr at 37 °C, without shaking. For quantification of biofilm formation, biofilms were stained with 0.1% crystal violet as described previously (Janssens et al., 2008). The minimum biofilm inhibitory concentration (MBIC) value was determined as the lowest concentration of AM404 required to inhibit biofilm formation.

To test the activity of AM404 against preformed *P. gingivalis* biofilms, 3‐day‐old biofilms were grown on peg lids as described above and were subsequently treated with 150 μl TSB containing AM404 (0–200 μM) for 24 hr at 37 °C. Next, pegs were washed with phosphate‐buffered saline (PBS) and the biofilms on the pegs were quantified by cell titer blue. Briefly, the peg lid was placed in a microtiter plate containing 200 μl of cell titer blue diluted 1/100 in PBS. After 24 hr of incubation in the dark at 37 °C, fluorescence was measured (λ_ex_: 535 nm and λ_em_: 590 nm) using the Synergy MX multimode reader (Biotek, Winooski, VT). The minimum biofilm reduction concentration (MBRC) was defined as the lowest concentration of AM404 able to kill bacteria growing in a biofilm.

### 2.4 Inhibition of biofilm formation on titanium disks by AM404

*P. gingivalis* biofilms were cultured on round titanium disks (commercially pure titanium, grade 2; height: 2 mm, width: 0.5 cm). Briefly, the disks were placed on the bottom of the wells of a 96‐well microtiter plate and were covered with 200 μl of a 1/10 diluted overnight culture containing 0 to 12.5 μM AM404. After 72 hr of incubation at 37 °C under static conditions, the disks were washed with PBS to remove nonadherent bacteria and were transferred to centrifuge tubes containing 1 ml PBS. Next, the disks were sonicated (45,000 Hz in a water bath sonicator [VWR USC 300‐T] for 10 min) and vortexed (1 min) to remove the bacteria from the disks. Serial dilutions of this bacterial solution were made and CFUs were determined by plating serial‐dilutions aliquots onto blood agar plates.

### 2.5 Activity of AM404 against established biofilms formed on titanium disks

To assess the ability of AM404 to disrupt preformed biofilms of *P. gingivalis* on titanium disks, 3‐day‐old biofilms were grown on the disks as described above. Thereafter, the disks were washed with PBS and were incubated with 200 μl of fresh TSB medium containing 0 to 50 μM AM404 for an additional 24 hr. Biofilm viability was quantified by CFU counts as described above.

### 2.6 Assessment of the viability of biofilms formed on titanium disks

The BacLight LIVE/DEAD bacterial viability staining kit (Molecular Probes, Invitrogen) was used to microscopically evaluate the viability of the biofilms formed on titanium disks. After incubation, the disks were washed with 1× PBS and were transferred to a LIVE/DEAD staining solution containing SYTO 9 and propidium iodide (prepared according to manufacturer's instructions). After 10 min of incubation at room temperature in the dark, the disks were washed again in 1× PBS and were mounted on a coverslip for imaging. The stained biofilm cells were visualized under a Zeiss Axio imager Z1 fluorescence microscope equipped with a EC Plan‐Neofluar 20× objective using the SYTO 9 (λ_ex_ = 483 nm; λ_em_ = 500 nm) and propidium iodide (λ_ex_ = 535 nm; λ_em_ = 617 nm) channels.

### 2.7 Cytotoxicity assays

HEK‐293 (human embryonic kidney cells), HepG2 (human hepatoma cells), IEC‐6 (rat intestinal cells), and Panc‐1 cells (pancreatic cancer cells) were purchased from ATCC (Rockville, MD, USA). These cell lines were chosen as they are extensively used in in vitro toxicity assays. Panc‐1 cells were cultured in Dulbecco's Modified Eagle Medium, HEK‐293 and HepG2 in Minimal Essential Medium, and IEC‐6 cells in Roswell Park Memorial Institute (RPMI) 1460 medium. All culture media were purchased form Life Technologies (Carlsbad, USA) and supplemented with 10% fetal calf serum, 2 mM L‐glutamine, 100 U/ml penicillin, and 100 μg/ml streptomycin, except for the RPMI medium, to which no L‐glutamine was added. Cell lines were cultured using standard cell culture techniques (37 °C, 5% CO_2_, and 95% humidity). Drug cytotoxicity testing was performed as described (Vriens et al., 2015). Briefly, following seeding, cells were treated for 24 hr with a control (1% DMSO) or a compound (0–253 μM). Subsequently, cell viability was determined by 3‐(4,5‐dimethylthiazol‐2‐yl)‐2,5‐diphenyltetrazolium bromide and expressed relative to the control treatment. The results were expressed as cytotoxic concentrations that induce 50% reduction in cell viability (CC_50_).

### 2.8 Membrane permeabilization assays

The ability of AM404 to permeabilize the outer and inner membranes of *P. gingivalis* was determined as previously described (Gerits et al., 2016), using the fluorescent dyes N‐phenyl‐1‐napthylamine (NPN, Sigma, USA) and SYTOX green (Invitrogen, USA), respectively; 1× the MIC of compound SPI031 (12.5 μM) was used as a positive control for outer membrane permeabilization because of its strong membrane permeabilizing properties (Gerits et al., 2016). Ten micrograms per milliliter melittin was used as a positive control for inner membrane permeabilization (Park et al., 2011).

### 2.9 Fluorescence microscopy

To visualize membrane damage induced by AM404, the membrane dye N‐(3‐triethylammoniumpropyl)‐4‐(p‐diethylaminophenyl‐hexatrienyl) pyridinium dibromide (FM 4‐64, Molecular Probes) was used. Briefly, exponential‐phase cells of *P. gingivalis* were incubated with DMSO (control) or 2× the MIC of AM404 for 30 min at 37 °C. Cells treated with 2× the MIC of triclosan (69.1 μM) were used as positive control. Cells treated with 2× the MIC of ciprofloxacin (1.9 μM), rifampicin (0.1 μM), and tetracycline (2.8 μM) were used as a negative control. Next, cells were collected by centrifugation and were stained with 10 μg/ml FM 4‐64 for 10 min at room temperature. Cells were visualized using a Zeiss Axio imager Z1 fluorescence microscope equipped with a EC Plan‐Neofluar 100× objective, using the FM 4‐64 channel (λ_ex_ = 506 nm; λ_em_ = 751 nm).

### 2.10 Statistical analysis and reproducibility of the results

Statistical analysis was performed by one‐way analysis of variance, followed by Dunnett's multiple comparison test. The *p* values <.001, <.01, and <.05 were considered to be statistically significant. All bacterial experiments were repeated at least 3 times. The cytotoxicity assay were performed in quadruplicate with four biological repeats.

## 3 RESULTS

### 3.1 AM404 shows antibacterial and antibiofilm activity against P. gingivalis

Previously, we screened the NIH repurposing library to identify new antibacterial compounds with activity against *P*. *gingivalis* (Gerits et al., 2017a,b). From this screening, three compounds were selected for further analysis: toremifene, zafirlukast, and AM404, which was further characterized in this study. In a first step, the activity of AM404 against different clinically important oral (*P*. *gingivalis*, *S*. *mutans*, P. intermedia, *F. nucleatum*, and *A. actinomycetemcomitans*) and nonoral pathogens (S. aureus, S. epidermidis, E. coli, P. aeruginosa, and *S*. Typhimurium) was evaluated. AM404 showed potent antibacterial activity against *P*. *gingivalis*, with a MIC and MBC value of 12.5 μM. AM404 showed slightly less activity compared to the antiseptic chlorhexidine (MIC = 6.4 μM). The compound was not active against all other pathogens examined (MIC and BIC > 100 μM). In addition, AM404 was effective against *P*. *gingivalis* biofilms grown on polystyrene pegs with MBIC and MBRC values of 12.5 μM. Next, a small‐scale structure–activity analysis was conducted including three analogs of AM404 to determine molecular components important for antibacterial activity (Table 1). This revealed that the unsaturated carbon chain may be crucial for activity of AM404, as evidenced by the inactivity of 4′‐hydroxystearanilide (MIC >200 μM). Furthermore, removal of the phenol group (R(+)‐methanandamide) or addition of a methoxy group to the phenol‐group (arvanil) led to a reduced antibacterial efficacy (MIC of 50 and 25 μM, respectively).

**Table 1:** MIC values of structural analogs of AM404

Compound	Structure	MIC (μM)
AM404	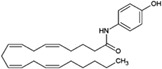	12.5
Arvanil	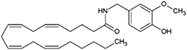	25
		
R(+)‐methanandamide	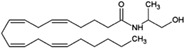	50
4′‐hydroxystearanilide	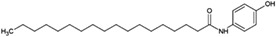	>200

*Note*. MIC = minimum inhibitory concentration.

### 3.2 AM404 is active against P. gingivalis biofilms grown on titanium disks

**Figure 1:**
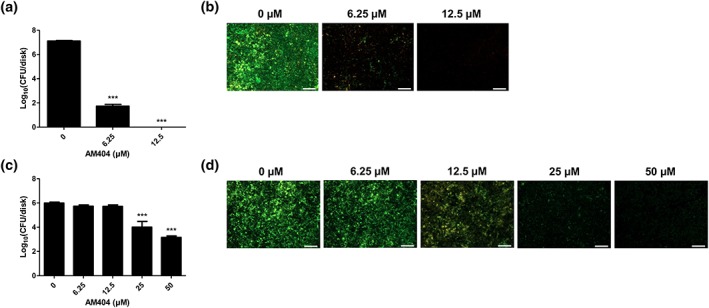
Effect of AM404 on *Porphyromonas gingivalis* biofilms formed on titanium disks. (a) Reduction of *P*. *gingivalis* biofilm formation in the presence of AM404, as determined by colony‐forming unit (CFU) counts. (b) Reduction of *P*. *gingivalis* biofilm formation in the presence of AM404, as determined by LIVE/DEAD staining. (c) Eradication of preformed biofilms of *P. gingivalis* by AM404, as determined by CFU counts. (d) Eradication of preformed biofilms of *P. gingivalis* by AM404, as determined by LIVE/DEAD staining. In (a) and (c), values are means ± SEM of three independent experiments. ^***^ *p* < .001 as compared to the untreated control. In (b) and (d), scale bars correspond to 100 μm. Images were processed with unsharp mask of Zen 2.0

Titanium is commonly used as an implant material for dental implants, as it has a high level of biocompatibility (Grosgogeat, Reclaru, & Dalard, 1999). Therefore, we tested AM404's activity against *P. gingivalis* biofilms grown on titanium disks. As shown in Figure 1a, the compound completely inhibits biofilm formation on titanium disks at a concentration of 12.5 μM. In addition, AM404 significantly reduces the number of CFUs in biofilms formed on these surfaces in a concentration‐dependent manner (Figure 1c). These results were further confirmed by LIVE/DEAD staining (Figure 1b,d). In the control treatment, green viable cells were predominant compared to the red dead cells. Treatment with AM404 resulted in a significant reduction of green cells. Only few red‐stained cells were observed after treatment, indicating that AM404 is able to fully clear established biofilms from surfaces.

### 3.3 AM404 displays limited cytotoxicity

The cytotoxicity of AM404 was evaluated against HEK‐293 (human embryonic kidney cells), HepG2 (human hepatoma cells), IEC‐6 (rat intestinal cells), and Panc‐1 cells (pancreatic cancer cells). As shown in Table 2, CC_50_ values for HEK‐293, HepG2, IEC‐6, and Panc‐1 cells are 67.77 ± 12.07, 42.16 ± 7.40, 73.29 ± 8.04, and 67.84 ± 5.91, respectively. These results show that AM404 is not toxic up to concentrations approaching 4× the MIC of AM404.

**Table 2:** Mean CC_50_ values ± SEM of AM404 against HEK‐293, HepG2, IEC‐6, and Panc‐1 cells (*n* = 4)

	CC_50_ (μM)
HEK‐293	67.77 ± 12.07
HEPG2	42.16 ± 7.40
IEC‐6	73.29 ± 8.04
Panc‐1	67.84 ± 5.91

*Note*. CC_50_ = cytotoxic concentration that induces 50% reduction in cell viability; HEK‐293 = human embryonic kidney cells; HepG2 = human hepatoma cells; IEC‐6 = rat intestinal cells; Panc‐1 cells = pancreatic cancer cells.

### 3.4 AM404 permeabilizes the inner and outer membrane of P. gingivalis

**Figure 2:**
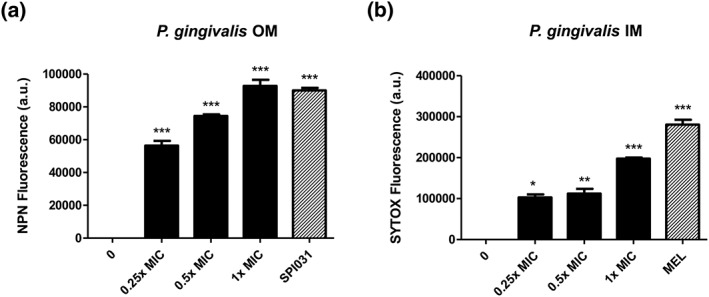
Effect of AM404 on (a) outer (OM) and (b) inner membrane (IM) permeabilization of *Porphyromonas gingivalis*, as assessed by N‐phenyl‐1‐napthylamine (NPN) and SYTOX green uptake, respectively; 1× the minimum inhibitory concentration (MIC) of SPI031 was used as a positive control for outer membrane permeabilization (Gerits et al., 2016). Ten micrograms per milliliter melittin (MEL) was used as a positive control for inner membrane permeabilization (Park et al., 2011). Data represent the means of three independent replicates ± SEM (^*^ *p* < .05, ^**^ *p* < .01, ^***^ *p* < .001)

To assess the mode of action of AM404 on the bacterial outer membrane, the hydrophobic fluorescent probe NPN was used. Under normal circumstances, NPN is excluded from the outer membrane. However, when the outer membrane is damaged, NPN can partition into the outer membrane, which results in increased fluorescence (Nikaido, 2003). As shown in Figure 2a, treatment of *P. gingivalis* cells with AM404 rapidly permeabilizes the outer membrane in a concentration‐dependent manner as observed by an increase in fluorescence. The nucleic acid stain SYTOX green was used to evaluate permeabilization of the inner membrane of *P*. *gingivalis* by AM404. SYTOX green is excluded from cells with intact inner membranes. However, when the inner membrane is disrupted, SYTOX green can enter the cytoplasm and bind with nucleic acids, which results in an increase in fluorescence (Roth, Poot, Yue, & Millard, 1997). As shown in Figure 2b, treatment with AM404 rapidly disrupts the integrity of the inner membrane in a concentration‐dependent manner. Outer and inner membrane permeabilization was induced by 1× the MIC of AM404 to the same extent as the positive controls SPI031 and melittin, respectively.

### 3.5 Microscopic visualization of membrane damage

**Figure 3:**
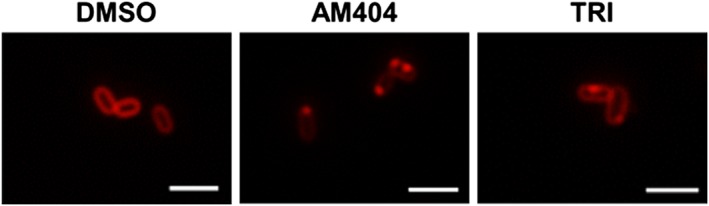
Visualization of antibiotic‐induced membrane damage using the fluorescent membrane dye FM 4‐64. Fluorescent images of *Porphyromonas gingivalis* cells after treatment for 30 min with dimethyl sulfoxide (DMSO; solvent control), 2× the minimum inhibitory concentration (MIC) of AM404 and 2× the MIC of triclosan (TRI). Scale bars correspond to 2 μm. Images were processed with unsharp mask of Zen 2.0

Disruption of membrane integrity by AM404 was further confirmed microscopically using the fluorescent membrane stain FM 4‐64 (Figure 3). Treatment with AM404 at 2× the MIC for 30 min results in nonhomogeneously stained membranes with clear membrane accumulations. Nonhomogeneously stained membranes are also observed after treatment of the cells with 2× the MIC of the membrane‐damaging antibiotic triclosan as a positive control. No such phenotype is observed after treatment of the cells with antibiotics with other modes of actions (ciprofloxacin [DNA synthesis], rifampicin [RNA synthesis], and tetracycline [protein synthesis]; Figure S1).

## 4 DISCUSSION

We demonstrated that AM404 shows strong antibacterial activity against *P*. *gingivalis*, with MIC, MBC, MBIC, and MBRC values of 12.5 μM. Strikingly, the activity of AM404 against planktonic and biofilm cultures appears to be similar, underlining the antibacterial potential of this compound. Thus, the biofilm lifestyle, including the presence of an extracellular matrix, did not affect the activity of AM404. No activity was found against other clinically important oral (*S*. *mutans*, P. intermedia, *F*. *nucleatum*, and *A*. *actinomycetemcomitans*) and nonoral pathogens (S. aureus, S. epidermidis, E. coli, P. aeruginosa, and *S*. Typhimurium), suggesting that the compound displays species‐specific activity. Future studies investigating the activity of AM404 against other strains of *P*. *gingivalis* can provide more clarity on this subject. A structure–activity analysis indicated that the unsaturated carbon chain may be essential for the compound's activity against *P*. *gingivalis*. A more thorough structure–activity analysis may shed more light on this relationship and is likely to lead to the identification of even more active compounds. We also demonstrated that AM404 can inhibit biofilm formation as well as eradicate preformed biofilms of *P. gingivalis* on both polystyrene and titanium surfaces. The latter underlines the potential of AM404 as an agent that can be used in the prevention and treatment of peri‐implantitis.

In recent years, more and more research has focused on finding new antibacterial compounds targeting a specific organism thereby reducing the effect on the commensal, resident flora. As for the oral cavity, different studies have published promising results on antimicrobial peptides specifically designed to target *S*. *mutans*, a key pathogen involved in caries (Eckert et al., 2006; Guo et al., 2015; Mai et al., 2011). Furthermore, two studies have previously identified antibacterial peptides specifically targeting *P*. *gingivalis* (Franzman et al., 2009; Suwandecha, Srichana, Balekar, Nakpheng, & Pangsomboon, 2015). In addition to their therapeutic effect, target‐specific antibacterials may be used as a tool to study the role of a particular pathogen in complex microbial communities such as biofilms found in the oral cavity (Frias‐Lopez, 2015; Guo et al., 2015). As such, in future research and in addition to studies validating the therapeutic effect of AM404, it would be interesting to use AM404 to further examine the role of *P*. *gingivalis* as a major pathogen in diseases such as periodontitis and peri‐implantitis. Naturally, for this to work, the activity of AM404 against *P. gingivalis* should be first tested in a mixed biofilm setup.

Several biological activities on eukaryotic cells have previously been reported for AM404, including activating the capsaicin receptor TRPV1, inhibiting COX, activating cannabinoid receptors, inhibiting cellular anandamide reuptake, and inhibiting NFAT activity and IκB kinase beta phosphorylation and activation (Bertolini et al., 2006; Caballero et al., 2015; Högestätt et al., 2005; Mallet et al., 2010; Ottani, Leone, Sandrini, Ferrari, & Bertolini et al., 2006). A recent study also identified AM404 as an antiviral agent that effectively inhibits replication of the dengue virus by binding with the NS4B protein (van Cleef, Overheul, Thomassen, Marjakangas, & van Rij, 2016). To our knowledge, no studies exist on the antibacterial activities of AM404 and its mode of action. In a first attempt to elucidate the antibacterial mode of action of AM404, its effect on the membrane of *P*. *gingivalis* was investigated. Interestingly, AM404 rapidly permeabilizes both outer and inner membranes, as evidenced by increased influx of NPN and SYTOX green into the cells. To visualize membrane damage caused by AM404, fluorescence microscopy images were obtained of *P*. *gingivalis* cells after treatment with AM404. Specific membrane accumulations were observed after treatment as compared to untreated cells. Similar accumulations were seen after treatment of the cells with the known membrane‐damaging agent triclosan. *P*. *gingivalis* is known to have atypical membrane structures as it possesses unique lipid A moieties that exhibit structural heterogeneity (Jain & Darveau, 2011). This aberrant lipid A also explains its resistance to polymyxin B, a membrane‐damaging agent frequently used as a last‐resort antibiotic for treatment of infections caused by Gram‐negative bacteria (Jain & Darveau, 2011; Kaye, Pogue, Tran, Nation, & Li, 2016). We therefore hypothesize that the selective activity of AM404 may be due to its specific binding to specific membrane structures of *P*. *gingivalis*, presumably via its highly hydrophobic carbon chain. In the future, additional tests should be performed to fully understand the molecular mechanisms behind the observed membrane damage.

In summary, we found that AM404 displays specific antibacterial and antibiofilm activity against *P. gingivalis*, presumably by damaging the membrane. To our knowledge, this is the first report of a nonpeptide antibacterial agent specifically targeting *P. gingivalis.* Peptide‐based antibacterial agents have some disadvantages such as high production costs, limited stability, and unknown toxicology (Marr, Gooderham, & Hancock, 2006), making AM404 a promising candidate for development of therapies targeting *P*. *gingivalis*. In future studies, it may be worth investigating the activity of AM404 against major virulence determinants of *P*. *gingivalis* as this is central to the pathogen's activity. In addition, the toxicity of AM404 against oral cell lines and its activity under in vivo conditions needs to be tested to fully establish its clinical importance. As AM404 is partly or fully responsible for the analgesic activity of paracetamol, this compound could be administered as a drug with a dual action, both reducing bacterial load and inflammatory pains associated with periodontal diseases (Borsani, Labanca, Bianchi, & Rodella, 2007; Högestätt et al., 2005; Mallet et al., 2010). Our findings together with the low cytotoxicity of the compound (Table 2) highlight the potency of this drug to be used for the development of new therapeutics for treatment of periodontitis and peri‐implantitis.

## Supporting information

Data S1. Figure S1. Fluorescence microscopy images of P. gingivalis cells stained with FM4‐64 after treatment with 2x the MIC of ciprofloxacin (CIP), rifampicin (RIF), and tetracycline (TET). Scale bars correspond to 2 μm. Images were processed with unsharp mask of Zen 2.0.
