# Supplementary figures and images for: Repurposing AM404 for the treatment of oral infections by Porphyromonas gingivalis
Source: Clin Exp Dent Res. 2017 Apr 7;3(2):69–76. doi: 10.1002/cre2.65 (PMC5719815; doi:10.1002/cre2.65)

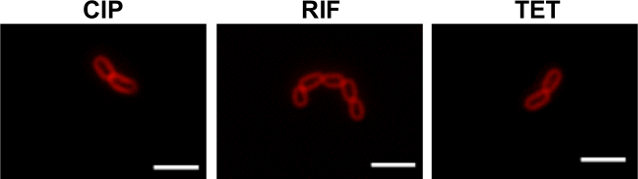

Supplement: Supplementary file 1 — Data S1. Figure S1. Fluorescence microscopy images of P. gingivalis cells stained with FM4‐64 after treatment with 2x the MIC of ciprofloxacin (CIP), rifampicin (RIF), and tetracycline (TET). Scale bars correspond to 2 μm. Images were processed with unsharp mask of Zen 2.0. [file CRE2-3-69-s001.tif]
